# Intradermal testing and allergen-specific IgE testing in dogs with eosinophilic bronchopneumopathy and healthy dogs

**DOI:** 10.3389/fvets.2026.1812293

**Published:** 2026-05-08

**Authors:** Hannah Gareis, Vanessa Hoerr, Ralf S. Mueller, Teresa Böhm, Laura Udraite-Vovk, Jelena Palić, Yury Zablotski, Bianka Schulz

**Affiliations:** 1 Clinic of Small Animal Internal Medicine, Ludwig Maximilian University of Munich, Munich, Germany; 2 Division of IDEXX Laboratories, Vet Med Labor GmbH, Kornwestheim, Germany

**Keywords:** allergy, atopy, bronchitis, canine, cough, EBP

## Abstract

**Background:**

Hypersensitivity reactions are considered as a potential etiology of canine eosinophilic bronchopneumopathy (EBP).

**Objectives:**

To investigate positive reactions in intradermal allergy testing (IDT) and allergen-specific serum immunoglobulin E (IgE) testing in dogs with EBP and healthy control dogs, and to compare the results between groups.

**Material and methods:**

Twenty-one dogs diagnosed with EBP and 22 healthy controls were included in the prospective case-control study. IDT with 41 allergens and an Fc-ε receptor-based serum test for allergen-specific IgE of 23 allergens were performed. Positive reactions were compared between groups using Pearson's Chi-squared test and false discovery rate correction for multiple testing. Agreement between the two test methods was evaluated with Cohen's Kappa test. The *p*-value was < 0.05 for all tests.

**Results:**

Dogs with EBP showed significantly more median positive reactions in IDT [8.0 (5.0–12.0)] compared to healthy control dogs [4.0 (1.0–8.0; *p* = 0.04)], but there was no difference in median positive reactions in serum IgE tests between dogs with EBP [11.0 (7.5–14.5)] and control dogs [11.0 (7.25–14.0; *p* = 0.36)]. Agreement between the results of both allergy testing methods was classified as minimal (κ = 0.14).

**Conclusion:**

Dogs with EBP are more likely to show positive reactions in IDT compared to healthy control dogs. However, similar findings in healthy controls and poor agreement with serum IgE results limit conclusions regarding clinical relevance. Thus, IDT and serum IgE results should be interpreted cautiously and in the context of clinical findings.

## Introduction

Canine eosinophilic bronchopneumopathy (EBP) is a chronic inflammatory disease characterized by infiltration of the lower airways and pulmonary interstitium with eosinophilic granulocytes especially in young adult dogs (1–3). The etiology of EBP is not yet completely understood, but hypersensitivity reactions have been discussed (2, 4). Studies have shown a selective increase in (cluster of differentiation) CD4^+^ T-cells and a decrease in CD8^+^ T-cells in the lower airways of affected dogs, but the ratio of CD4^+^/CD8^+^ T-cells returned to normal under glucocorticoid therapy (4, 5). The increased ratio of CD4^+^/CD8^+^ cells suggests that, similar to eosinophilic lung disease in humans, a Th2-mediated immune response may be responsible for the bronchopulmonary changes (6–8). In a previous study, intradermal allergy testing (IDT) with 48 allergens was performed in 12 dogs with untreated EBP. In that study, 4/12 dogs showed positive and six dogs showed negative reactions, while tests in two patients could not be evaluated. However, no control group was included for comparison in that study (4). In a case series documenting IDT results in three dogs with EBP, none of the dogs showed any positive reactions (3). Due to an incomplete understanding of the underlying disease mechanisms that drive EBP, treatment typically aims at suppression of the inflammatory response and therefore involves glucocorticoids. Prolonged use of these drugs is often required to control clinical signs (2).

Identifying allergens potentially involved in the etiology of EBP could improve understanding of the pathogenesis of canine EBP, and possibly offer a chance to pursue allergen avoidance as a potential glucocorticoid-sparing therapeutic option in canine EBP in the future. In addition, allergen (-specific) immunotherapy (AIT) could be a treatment option if relevant allergens can be identified.

The aim of the study was to investigate positive reactions in IDT and allergen-specific serum IgE testing in dogs with EBP and in young healthy dogs from a research setting and compare the results between groups.

## Materials and methods

The study was approved by the Government of Upper Bavaria, Germany (number: 55.2-1-54-2532-117-201). The investigation was conducted as a prospective case-control study at the Clinic of Small Animal Internal Medicine of Ludwig Maximilian University (LMU) of Munich, Germany. Written consent was given by all dog owners.

### Study participants

Client-owned dogs with respiratory clinical signs suggestive of EBP, such as a chronic cough, exercise intolerance, increased respiratory effort and respiratory distress, presented to the LMU Clinic of Small Animal Medicine for diagnostic workup, were eligible for the study. For further evaluation, hematology, serum biochemistry and thoracic radiographs in two views (laterolateral and ventrodorsal) were obtained. Serum was stored at −80 °C before being shipped for allergen-specific and total serum immunoglobulin E (IgE) testing. Patients were anesthetized using various protocols according to ASA classification and intubated with a sterile endotracheal tube. Intradermal allergy skin testing, bronchoscopy and bronchoalveolar lavage (BAL) were performed with 5–10 ml of sterile, isotonic saline solution (0.9%, B. Braun Vet Care) applied over the working channel of the endoscope in 2–3 aliquots in representative areas of both lung sides and retrieved via a mechanical vacuum device. Collected samples were pooled, centrifuged and smears were stained with modified Wright's stain for cytological evaluation. The cell count of the bronchoalveolar lavage fluid (BALF) was determined in each sample before centrifugation. Cytological examination of all smears was carried out by a board-certified clinical pathologist. For this purpose, at least two direct smears and one to two cytospin preparations were examined in each case. Per slide, 100 inflammatory cells were differentially counted in several microscopic fields. In addition, each BALF was submitted for aerobic bacterial culture to the LMU Institute of Infectious Diseases and Zoonoses, Munich, Germany. Additional tests (PCR test for pulmonary parasites or *Mycoplasma* spp.) were performed upon discretion of the primary clinician.

Inclusion criteria for dogs with EBP were the presence of respiratory signs indicative of EBP, detection of eosinophilic inflammation in BALF, defined as >14 % eosinophils on cytology, and the exclusion of other causes of eosinophilic airway inflammation, with all performed diagnostic evaluations required to support a diagnosis of EBP. Dogs were also not included if they were not stable enough for a diagnostic workup or if cardiac or pleural space disease or respiratory disease other than EBP was detected, based on clinical, radiographic or BALF-examination findings. Lung worm infection was excluded by *Angiostrongylus vasorum* serum antigen testing (11/21), Baermann-Wetzel fecal examination (13/21), and/or diagnostic deworming (10/21). Bacterial infection was excluded by BALF-culture in all dogs and PCR-testing was performed for selected agents (*Mycoplasma* sp, *Bordetella bronchiseptica, Angiostrogylus vasorum*) if indicated.

The control group consisted of healthy research dogs. that were all kept under the same environmental conditions in a research kennel. All control dogs were current on vaccinations and deworming. Inclusion criteria for the control dogs were an unremarkable clinical examination and the absence of respiratory or dermatological signs for at least 6 months prior to inclusion.

All dogs were not permitted to receive antihistamines or systemic glucocorticoids for at least four, and inhaled glucocorticoids for at least 2 weeks prior to allergy testing, respectively.

### Owner questionnaire

All owners of the EBP-affected dogs completed a standardized questionnaire that included questions about clinical signs, as well as environmental, dietary and husbandry conditions. The owners were also asked to report whether they smoked in the dog's environment.

### Allergen-specific and total serum IgE testing

Serum samples were collectively shipped to Heska Diagnostic Laboratory (Fribourg, Switzerland) for allergen-specific and total IgE testing. Allergen-specific IgE was determined for 23 allergens (Table 1) and, additionally, total IgE was determined. First, a test for the presence of anti-cross reactive carbohydrate determinants (CCD) IgE was performed. In samples negative for CCD antibodies, a commercial ELISA (Heska Allercept Panel, Heska AG, Fribourg, Switzerland) was performed for 23 allergens, using a recombinant α-chain of the high-affinity IgE-receptor (FcεRIα). Samples positive for anti-CCD IgE were tested with the same ELISA after the serum had been treated with a Heska proprietary CCD blocker. Results were obtained by converting the optical densities for each allergen into HERBU (Heska Epsilon Receptor Binding Units). The results were graded into four classes. HERBU values < 10 were considered negative; positive classes were divided into low (11–25), moderate (26–50), and high (>51).

**Table 1:** Allergens used in serum IgE testing.

Subgroup	Latin name	English name
Mites	*Dermatophagoides pteronyssinus*	Dust mite
	*Dermatophagoides farinae*	Dust mite
	*Tyrophagus putrescentiae*	Mold mite
	*Acarus siro*	Grain mite
Grasses	*Dactylis glomerata*	Orchard grass
	*Phleum pratense*	Timothy grass
	*Cynodon dactylon*	Bermuda grass
	*Poa pratensis*	Blue grass
	*Lolium perenne*	Ryegrass
	*Festuca pratensis*	Meadow fescue
	*Holcus lanatus*	Velvet grass
Herbs	*Artemisia vulgaris*	Mugwort
	*Chenopodium album*	White goosefoot
	*Plantago lanceolata*	Ribwort
	*Rumex acetosella*	Red sorrel
	*Ambrosia artemisfolia*	Common ragweed
Trees	*Corylus avellana*	Common hazel
	*Betula populifolia*	Gray birch
	*Alnus* sp.	Alder
	*Quercus* sp.	Common oak
	*Fraxinus* sp.	Ash
Others		Flea saliva
	*Malassezia*	

### Intradermal allergy testing (IDT)

IDT was performed as previously described (9) by a board-certified dermatologist, as well as dermatology residents, who were experienced in the procedure. 41 allergens (Artu Biologicals Europe B.V., Lelystad, The Netherlands), each in 0.05 ml of allergen solution, were injected intradermally into the clipped lateral chest or abdominal wall of all dogs (Table 2). The allergen concentration was 200 Noon units (NU)/ml for pollen antigens, 100 NU/ml for mite antigens, 1,000 NU/ml for flea saliva antigen, and 100 μg/ml for Malassezia antigen, respectively. As a positive control 0.1 % histamine was injected; the dilution solution of the allergens (phosphate-buffered saline solution with 0.47 % phenol) served as a negative control. Reactions were evaluated after 15 and 25 min. The local reactions were graded into five categories from 0 to 4, based on diameter, induration and erythema of the wheals (9). Reactions of 0 and 1 were defined as negative; reactions from 2 to 4 were defined as low- (2), moderate- (3) or high-grade (4) positive reactions.

**Table 2:** Allergens used in intradermal testing.

Subgroup	Latin name	English name
Mites	*Dermatophagoides pteronyssinus*	Dust mite
	*Dermatophagoides farinae*	Dust mite
	*Tyrophagus putrescentiae*	Mold mite
	*Acarus siro*	Grain mite
	*Neotrombicula autumnalis*	Harvest mite
Insects	*Blatta orientalis*	Cockroach
	*Tabanus*	Cattle fly
	*Musca domestica*	Common housefly
	*Aedes*	Tropical mosquito
	*Culex*	Common mosquito
	*Culicoides*	Midge
Grasses	*Dactylis glomerata*	Orchard grass
	*Cynodon dactylon*	Bermuda grass
	*Poa pratensis*	Blue grass
	*Lolium perenne*	Ryegrass
	*Holcus lanatus*	Velvet grass
	*Elymus repens*	Couch grass
	*Agrostis capillaris*	Common bent
	*Alopecurus pratensis*	Meadow foxtail
Herbs	*Artemisia vulgaris*	Mugwort
	*Chenopodium album*	White goosefoot
	*Plantago lanceolata*	Ribwort
	*Rumex acetosella*	Red sorrel
	*Ambrosia artemisfolia*	Common ragweed
	*Solidago*	Goldenrod
	*Parietaria*	Glasswort
	*Brassica napus*	Rapeseed
Trees	*Corylus avellana*	Common hazel
	*Fagus*	Beech
	*Populus alba*	Silver poplar
Fungi	*Alternaria*	Black mold fungus
	*Aspergillus*	Mold fungus
	*Cladosporium*	Black mold fungus
Others		Flea saliva
	*Malassezia*	
		Sheep's wool
		Goose feathers
		Epithelia mix
		Tree pollen mix 1
		Tree pollen mix 2
		Herbal pollen mix

### Statistics

The statistical evaluation was performed using R version 3.6.3. (10). Data was tested for normality with the Shapiro-Wilk test, and presented as mean ± standard deviation for normally distributed data or median with interquartile range (IQR) for not normally distributed data. Since data was not normally distributed (scores), the Wilcoxon rank-sum test was used for comparison of groups. Categorical data was analyzed with the Pearson Chi-squared test. The level of significance for all tests was set at *p* ≤ 0.05. False discovery rate correction for multiple testing was performed. Agreement between both methods (IgE test and IDT) was evaluated with Cohen's Kappa test. Kappa values < 0 indicated no agreement, 0–0.20 minor agreement, 0.21–0.40 fair agreement, 0.41–0.60 moderate agreement, 0.61–0.80 substantial agreement and 0.81–1 almost perfect agreement (11).

## Results

### Study population

Twenty-one dogs with EBP were included in the study: 11 males (seven castrated) and 10 females (seven spayed). No particular breed was overrepresented: mixed-breed (*n* = 5), Siberian Husky (*n* = 2), Old German Shepherd (*n* = 2), Rottweiler (*n* = 1), Tibet Terrier (*n* = 1), Flat Coated Retriever (*n* = 1), Mudi (*n* = 1), Husky (*n* = 1), Labradoodle (*n* = 1), Sheltie (*n* = 1), Boston Terrier (*n* = 1), Australian Shepherd (*n* = 1), Golden Retriever (*n* = 1), Bernese Mountain Dog (*n* = 1) and Akita (*n* = 1). The mean age at the time of diagnosis was 2 years (± 3.17 years). Twenty-two healthy dogs from a research kennel were included as a control group. These consisted of 14 Beagles and eight Foxhound-crossbreeds, 11 of them female (not spayed) and 11 male (six castrated). The mean age of the control dogs was 2 years (± 1.25 years), which comprised no significant age difference compared to the EBP group (*p* = 0.41).

### Owner questionnaire

The owners of 3/21 (14.2 %) dogs with EBP reported smoking in the presence of their dog, and 8/21 (38.1 %) had a fireplace or stove at home. Companion animals were present in the household of 18/21 (85.7 %) EBP dogs and consisted of dogs [13/21 (61.9 %)], cats [7/21 (33.3 %)], small mammals [2/21 (9.5 %)], birds [2/21 (9.5 %)] and large animals [2/21 (9.5 %)]. A seasonal impact on clinical signs was described in 38.1 % (8/21) of the dogs: half of the dogs showed clinical signs primarily during the summer, whereas the other half exhibited clinical signs mainly in the winter.

### Clinical findings

The most common respiratory clinical sign in the EBP group was coughing, in 85.7 % (18/21) of the dogs. In addition, the dogs with EBP showed exercise intolerance (57.1 %, 12/21), sneezing and nasal discharge (19.0 %, 4/21), dyspnea (14.2 %, 3/21), and reverse sneezing (9.5 %, 2/21). To assess a potential hypersensitivity component or allergic condition in the context EBP, clinical signs consistent with food or environmental allergies, including dermatologic abnormalities and gastrointestinal signs, were systematically evaluated. In this context, dogs with EBP showed signs such as vomiting and gagging (47.6%, 10/21), diarrhea (9.5%, 2/21), and dermatologic abnormalities including pruritus and alopecia (9.5%, 2/21). None of the clinical signs were significantly associated with eosinophil count in peripheral blood or BALF.

### Allergen-specific and total serum IgE measurements

IgE testing was performed in 19/21 dogs with EBP and in all healthy dogs. The total IgE in dogs with EBP showed a median of 68.30 μg/ml (44.23–306.88) vs. 132.25 μg/ml (94.22–284.23) in the control group. This difference was not significant (*p* = 0.23). Dogs with EBP showed a median of 11 (7.5–14.5) and the control group a median of 11 (7.25–14.0) positive reactions, respectively. No significant difference was detected between the groups (*p* = 0.36). The results for all allergen reactions are shown in Table 3.

**Table 3:** Number of positive results in serum IgE testing in dogs with EBP compared to healthy control dogs.

Subgroup	Allergen	EBP (*n* = 19)	Control (*n* = 22)	*p*- value
Mites	*Dermatophagoides pteronyssinus* (dust mite)	17	22	0.47
	*Dermatophagoides farinae* (dust mite)	19	22	0.83
	*Tyrophagus putrescentiae* (mold mite)	16	19	0.87
	*Acarus siro* (grain mite)	18	22	0.66
Grasses	Orchard grass	10	11	>0.99
	Timothy grass	4	6	0.87
	Bermuda grass	10	9	0.83
	Blue grass	5	12	0.41
	Ryegrass	8	11	0.83
	Meadow fescue	14	17	0.83
	Velvet grass	5	7	0.83
Herbs	Mugwort	5	7	0.83
	White goosefoot	5	6	0.95
	Ribwort	8	9	0.99
	Red sorrel	12	15	0.83
	Common ragweed	7	4	0.66
Trees	Common hazel	8	8	0.92
	Gray birch	8	4	0.47
	Alder	8	6	0.83
	Common oak	9	2	0.90
	Ash	2	1	0.83
Others	Flea saliva	7	0	0.07
	*Malassezia*	3	0	0.47

No significant differences after correction for multiple testing were detected (only allergens with at least one positive reaction are listed).

### Intradermal testing

IDT was performed in 20/21 dogs with EBP and all healthy dogs. Dogs with EBP showed a median of 8.0 (5.0–12.0) positive reactions to the allergens tested; the control group had a median of 4.0 (1.0–8.0) positive reactions. Dogs with EBP showed significantly more overall positive reactions than dogs in the control group (*p* = 0.04). There was no significant difference between the number of positive reactions to the individual allergens of dogs with EBP compared to the healthy control group. The results of IDT in the dogs with EBP compared to the healthy control dogs are shown in Table 4.

**Table 4:** Number of positive results in intradermal testing in dogs with EBP compared to healthy control dogs.

Subgroup	Allergen	EBP (*n* = 20)	Controls (*n* = 22)	*p*-value
Mites	*Dermatophagoides pteronyssinus* (dust mite)	11	8	0.89
	*Dermatophagoides farina* (dust mite)	15	11	0.81
	*Tyrophagus putrescentiae* (mold mite)	13	12	0.99
	*Acarus siro* (grain mite)	15	10	0.81
	*Neotrombicula autumnalis* (harvest mite)	12	8	0.82
Insects	Cockroach	0	1	>0.99
	Cattle fly	14	7	0.44
	Common housefly	1	0	0.96
	Tropical mosquito	4	0	0.99
	Common mosquito	4	4	0.96
	Midge	7	5	0.96
Grasses	Orchard grass	2	5	0.95
	Bermuda grass	0	3	0.86
	Blue grass	2	4	>0.99
	Ryegrass	2	0	0.86
	Velvet grass	3	2	0.99
	Couch grass	1	1	>0.99
	Common bent	3	4	>0.99
	Meadow foxtail	2	4	0.99
Herbs	Mugwort	2	1	0.99
	White goosefoot	1	1	>0.99
	Ribwort	7	1	0.44
	Red sorrel	4	3	>0.99
	Common ragweed	1	4	0.99
	Goldenrod	0	1	>0.99
	Glasswort	4	6	>0.99
	Rapeseed	4	4	>0.99
Trees	Common hazel	0	1	>0.99
	Beech	4	0	0.67
Fungi	*Alternaria*	1	0	0.96
	*Aspergillus*	1	0	0.96
	*Cladosporium*	1	0	>0.99
Others	*Malassezia*	2	1	0.99
	Flea saliva	4	1	0.81
	Sheep's wool	2	1	0.99
	Goose feathers	3	4	>0.99
	Tree pollen mix 1	6	9	0.96
	Tree pollen mix 2	5	5	>0.99
	Herbal pollen mix	5	1	>0.99

No significant differences after correction for multiple testing were detected (only allergens with at least one positive reaction are listed).

### Agreement between serum IgE measurements and IDT

The Cohen's Kappa between all results of the IDT and serum IgE measurements showed minimal agreement (κ = 0.14). Similarly, there was only minimal agreement for both tests within both groups, in the EBP group (κ = 0.13) and in the control group (κ = 0.14).

## Discussion

The study aimed to investigate serum IgE and IDT in dogs with EBP and compare the results with young healthy control dogs to gain a better understanding of the pathophysiology of the disease and to evaluate possible additional treatment options.

Dogs with EBP show significantly more positive reactions in IDT compared to healthy controls. This result matches that of previous studies in which a higher ratio of CD4:CD8 lymphocytes in the BALF of dogs with EBP was detected compared to healthy dogs, suggesting a CD4-mediated Th2 immune response (4, 5, 12). Positive IDT results indicate the activation of cutaneous mast cells and the presence of allergen-specific IgE which supports a Th2 immune response. Increased levels of CD4 lymphocytes and a Th2-mediated immune response have also been demonstrated in allergic respiratory tract diseases in humans and mice (6, 8, 13–15), supporting results of this study investigating EBP in dogs.

In other aspects, the results of the current study differ from previous studies in which IDT was performed in dogs with EBP: in one study reporting IDT results in 12 dogs with EBP, only 33 % of the dogs showed positive reactions, while 50 % of the dogs did not react to any allergen, and tests in two patients could not be evaluated (4). In another case series, ID tests of three dogs with EBP all showed negative reactions (3). The small number of patients, potential pre-treatment with corticosteroids or antihistamines, differences in the number and type of allergens tested and the lack of standardization could explain the low number of positive reactions in these previous reports compared to the present study. In addition, both earlier investigations lacked a control group. However, as no individual allergen in IDT was significantly more frequently positive in dogs with EBP compared to the control group, these findings may indicate an overall increased general sensitization in dogs with EBP relative to healthy controls. Nevertheless, no specific allergic trigger could be identified. This supports the multifactorial nature of EBP and highlights that IDT alone is not suitable for determining the underlying cause of the disease.

In contrast to the results of the IDT, there were no significant differences between healthy dogs and dogs with EBP in terms of total serum IgE levels or the number of positive reactions in allergen-specific IgE testing. In a prior study evaluating atopic dogs, it was shown that serum-specific IgE levels were also not significantly different between patients and healthy control dogs (16), supporting similar findings of the current study In a study investigating cats with feline lower airway disease (FLAD), those with chronic sterile mixed airway inflammation showed significantly higher allergen-specific IgE compared to healthy cats. However, cats with predominantly eosinophilic and neutrophilic airway inflammation showed no significant difference in allergen-specific IgE levels when compared to healthy controls (17). In the present study, only dogs with eosinophilic airway inflammation were examined, therefore, comparisons with other disease complexes and species can only be made to a limited extent.

In accordance with previous investigations, most of the healthy dogs in the control group of the present study showed positive reactions in both tests comparable to dogs with EBP (18–20). This finding contrasts with the conventional assumption of an allergen-driven pathogenesis of EBP. Causes may be asymptomatic sensitization without clinical relevance or, less likely, an irritant reaction in the IDT (18, 19, 21). It is known that the exposure time of the triggering allergens, as well as season and patient age, can influence serum IgE levels (22–24). In addition, in the present study the control group consisted of two different breeds only, which could have affected the results, as a previous study showed a genetic influence on serum IgE concentration in different breeds (25). In a previous investigation, positive allergen-specific serum reactions were seen more commonly in healthy cats compared to cats with FLAD (26), which shows accordance to the results in dogs with chronic airway disease. Additionally, the relatively small sample size may have contributed to the findings. Overall, the high number of positive reactions in both tests in the control group may reflect clinically irrelevant sensitization rather than true disease-associated hypersensitivity. Therefore, no specific allergen could be identified as a trigger for EBP. This finding supports the multifactorial nature of the disease and suggests that serum IgE testing and IDT have limited value in identifying causative allergens in dogs with EBP.

In the present study, serum samples were first tested for anti-CCD IgE and, if this was present, were treated with a CCD blocker prior to allergen testing. This procedure leads to improvement in the agreement between IDT and IgE measurement, as demonstrated in a previous investigation in atopic dogs (27). However, the agreement between the two test systems was lower in the current study as compared to the previous study (27). In the current study, positive reactions were more common in serum testing than in IDT. In studies in atopic dogs, a weak agreement was observed between the two test methods, with an increased number of positive results in serum testing (28, 29). One possible explanation is the concentration of the different allergens used in IDT. There is no standardized procedure for IDT and studies on threshold concentrations have been inconclusive (19, 30). Another explanation for the differences in results between the two test methods could be the fact that, in IDT, the response of IgE attached to mast cells is evaluated, while free IgE is measured in the serum test (21, 23). In addition, it cannot be excluded that variations in allergen extracts, such as different quality and lack of standardization in purity, also play a role, resulting in discrepancies between test results.

The minimal agreement between the results of IDT and serum IgE testing in this study suggests that the two methods may not always produce consistent results, and may have different diagnostic values in patients with EBP. A similar result was seen in a study investigating the agreement between allergy testing via serum IgE and IDT in cats with FLAD (26). This highlights the need for a comprehensive diagnostic approach that takes into account the limitations and potential biases of the different test methods. It also indicates the likelihood that the two test methods cannot be used interchangeably and that both must be carried out to obtain full information about potential allergic sensitization in EBP patients, as they may reflect different aspects of immunologic sensitization

Reactions to mites were very common in the EBP patients; however, they were equally common in the healthy control dogs. Sensitization to dust mites is common in atopic and healthy dogs, as previous studies with IDT and IgE testing have revealed (28–30). High rates of sensitization can be explained by the fact that the housing, bedding and hair of dogs are commonly contaminated with dust mite antigens (31). Sensitization to storage mites, induced by allergen-contaminated dog food and environmental exposure, is also common in atopic and healthy dogs (16). It is well known from previous studies comparing atopic and healthy dogs that exposure and sensitization are not necessarily associated with the development of clinical signs, which could also be shown in the present study. Therefore, a decision in favor of allergen (-specific) immunotherapy should not be based on allergy test results alone in patients with EBP, but should also take clinical and environmental factors into account when compiling an individual treatment plan for symptomatic patients. Similar results and conclusions have been made in cats with FLAD (26, 32).

The present study has several limitations. The group size was relatively small, influencing the statistical power of the results. In addition, the control group in this study consisted of a research colony of two breeds of dog of young age, which means that the dogs were not breed- and age-matched. It has been shown that both breed and age are factors that may influence serum IgE concentrations (22–25), a fact which should be considered when interpreting the results of this study. However, obtaining age- and breed-matched control dogs for IDT, which requires sedation, is challenging. In addition, the control group was maintained under uniform environmental conditions whereas dogs with EBP were client-owned and exposed to heterogeneous environments and allergens. As allergen sensitization is influenced by environmental and genetic factors, these differences may have affected the observed results and should be considered when interpreting the findings. Likely additional factors, such as housing, nutrition, and allergen exposure, also have an influence on allergy test results and should be considered in future studies. A further limitation of this study is the use of the number of positive reactions as an outcome measure, which may oversimplify the biological and clinical relevance of allergen sensitization, as it does not account for allergen type, level of exposure, or clinical significance, and therefore limits conclusions regarding causality. It remains unclear whether allergens causing positive reactions in allergy tests are in fact causally related to EBP. This study focused on the role of allergen sensitization in patients with EBP. However, there are likely to be other factors that contribute to the development and progression of this respiratory condition. Environmental factors, genetic predisposition and co-existing diseases, for example, could all play a role in the pathogenesis of EBP. This information could help to improve development of more personalized and effective treatment strategies for dogs with this condition.

## Conclusion and relevance

Dogs with EBP are more likely to show reactions in IDT, suggesting a possible role of hypersensitivity mechanisms. However, there is a great similarity with allergy testing results in healthy dogs; in addition, the agreement between results of IDT and serum IgE testing is only minor. Thus, the clinical significance of allergy testing in EBP remains uncertain. In conclusion, positive reactions should be interpreted with caution and always within the context of diagnostic findings. IDT and serum IgE testing cannot be used interchangeably. Further research is needed to to develop a more comprehensive understanding of the complex mechanisms underlying EBP and the role of allergy testing in its diagnostic assessment.

## Data Availability

The raw data supporting the conclusions of this article will be made available by the authors, without undue reservation.
